# Trends in new HIV diagnoses and factors contributing to late diagnosis among migrant populations in EU/EEA countries, 2014 to 2023

**DOI:** 10.2807/1560-7917.ES.2024.29.48.2400759

**Published:** 2024-11-28

**Authors:** Juliana Reyes-Urueña, Giorgia Stoppa, Federica Pizzolato, Marieke J van der Werf, Charlotte Deogan, Vítor Cabral-Veríssimo, Helena Cortes-Martins, Jessika Deblonde, Asuncion Diaz, Victoria Hernando, Erna Milunka-Kojic, Joël Mossong, Kate O'Donnell, Eline Op de Coul, Chrysa Tsiara, Lilian van Leest, Dominique Van Beckhoven, Maria Wessman, Robert Whittaker, the EU/EEA HIV network, ECDC/WHO HIV Surveillance Network members, Giedrė Aleksienė, Mary Archibald, Maria Axelsson, Birgit van Benthem, Joana Bettencourt, Tatjana Nemeth Blazic, Pierre Braquet, Henrikki Brummer-Korvenkontio, Alexandra Bražinová, Anneli Carlander, Françoise Cazein, Helena Cortes Martins, Jessika Deblonde, Anna Demetriou, Lena Dillner, Asuncion Diaz, Ziad El-Khatib, Jevgenia Epstein, Georgios Ferentinos, Ágnes Galgóczi, Anna Margret Gudmundsdottir, Barbara Gunsenheimer-Bartmeyer, Patrick Hoffmann, Derval Igoe, Irene Kászoni-Rückerl, Mirjana Lana Kosanovic Licina, Irena Klavs, Šarlote Konova, Erna Milunka Kojic, Tanja Kustec, Lilian van Leest, Elaine Lautier, Kirsi Liitsola, Florence Lot, Jackie Maistre Melillo, Marek Malý, Mariana Mardarescu, Joël Mossong, Marta Niedźwiedzka-Stadnik, Kate O’Donnell, Eline Op de Coul, Jurgita Pakalniškienė, Dimitra Paraskeva, Pedro Pinto Leite, Kristi Rüütel, Magdalena Rosinska, George Siakallis, Barbara Suligoi, Maria Elena Tosti, Vítor Cabral Veríssimo, Esther Walser-Domjan, Maria Wessman, Robert Whittaker, Elena Xenofontos, Hana Zákoucká, Natig Zulfugarov

**Affiliations:** 1 European Centre for Disease Prevention and Control (ECDC), Stockholm, Sweden; 2 Unit of Biostatistics, Epidemiology and Public Health, Department of Cardiac, Thoracic, Vascular Sciences and Public Health, University of Padova, Padua, Italy; 3 Division of Epidemiology and Statistics, Directorate-General of Health, Lisbon, Portugal; 4 Department of Infectious Diseases, National Institute of Health Dr Ricardo Jorge, Lisbon, Portugal; 5 Epidemiology of Infectious Diseases, Public Health and Surveillance, Sciensano, Brussels, Belgium; 6 Unidad de Vigilancia de VIH, ITS y Hepatitis B y C, Centro Nacional de Epidemiología, CIBERINFEC, Instituto de Salud Carlos III, Madrid, Spain; 7 Division of Infectious Diseases, Landspítali Haskolasjúkrahus, Reykjavik, Iceland; 8 Division de l’inspection sanitaire, Ministère de la Santé et de la Sécurité Sociale, Luxembourg, Luxembourg; 9 Health Protection Surveillance Centre, Dublin, Ireland; 10 National Institute for Public Health and the Environment (RIVM), Bilthoven, the Netherlands; 11 HIV/AIDS Surveillance Department, National Public Health Organization, Athens, Greece; 12 Unit for Epidemiological Monitoring, Public Health Agency of Sweden, Stockholm, Sweden; 13 Department of Infectious Disease Epidemiology and Prevention, Statens Serum Institut, Copenhagen, Denmark; 14 Section for respiratory, blood-borne and sexually transmitted infections, Department of Infection Control and Vaccines, Norwegian Institute of Public Health, Oslo, Norway; 15 The members of the EU/EEA HIV network are listed under Collaborators; * These authors contributed equally to this work.

**Keywords:** HIV infections, epidemiology, population surveillance, migrants, Healthcare, Delayed Diagnosis

## Abstract

We analysed trends in new HIV diagnoses and factors contributing to late diagnosis among migrants in countries in the European Union (EU)/European Economic Area (EEA) from 2014 to 2023. Of the total reported HIV diagnoses, 45.9% were in migrants, with 13.3% born in EU/EEA countries and 86.7% in non-EU/EEA countries. Late diagnosis was observed in 52.4% of migrants, particularly among non-EU/EEA migrants with heterosexual transmission, regardless of sex. Improved HIV prevention and testing strategies are essential for at-risk migrant populations.

Migrants, defined as people born outside of the country in which they reside, are a key population affected by HIV in European Union (EU)/European Economic Area (EEA) countries. In 2023, migrants accounted for 47.9% (11,837/24,731) of all HIV diagnoses in the EU/EEA [1].

Migrants are a diverse group, with various drivers of migration and various HIV risk factors. As the HIV epidemic evolves, public health strategies must adapt to shifting epidemiological trends, refining approaches to prevention, testing, and treatment to achieve the Sustainable Development Goals set for 2030. To guide these programmes, we aimed to assess trends in new HIV diagnoses among migrants in EU/EEA countries from 2014 to 2023, focusing on sociodemographic factors associated with late diagnosis.

## HIV diagnoses among migrants in EU/EEA countries

Between 2014 and 2023, a total of 247,733 HIV diagnoses were reported by 30 EU/EEA countries to the European Centre for Disease Prevention and Control through the European Surveillance System. For this analysis, we only included new HIV diagnoses, leading to the exclusion of 99,909 cases from 12 countries unable to classify diagnoses as either new or previously positive. Consequently, we also excluded all previously positive diagnoses (n = 24,498) reported by the remaining 19 countries, leaving a total of 123,326 new HIV diagnoses which could be analysed.

We established two categories related to region. The first, ‘region of origin’ defines migrants’ birth region. Region of origin was categorised based on UNAIDS designation and divided into non-EU/EEA and EU/EEA countries. The second category, ‘EU/EEA subregions’, defines sub-regions for the reporting countries within the EU/EEA. A list of countries by region is appended in the Supplement.

Among the diagnosed people with known region of origin (n = 103,416, or 83.9% of the study sample), 45.9% (n = 47,473) were migrants, i.e. born outside of the EU/EEA country where they were diagnosed. Characteristics of these cases are presented in Table 1 and, for purposes of comparison, cases among non-migrants are also presented. Among migrants in this study sample, 13.3% (n = 6,337) were born in another EU/EEA country and 86.7% (n = 41,136) in non-EU/EEA countries. By region, 44.7% (n = 21,232) were born in Sub-Saharan Africa, 13.9% (n = 6,601) in Latin America and the Caribbean, and 11.2% (n = 5,335) in eastern Europe, with smaller proportions in central Europe, western Europe, South/South-East Asia and other regions. The western EU/EEA subregion reported 81.0% (n = 5,133) of migrants originating from other EU/EEA countries and 72.9% (n = 29,995) of migrants originating from non-EU/EEA countries (Table 1).

**Table 1:** Sociodemographic characteristics of migrant and non-migrant populations diagnosed with HIV, EU/EEA, 2014–2023 (n = 123,326)

	Cases with known region of origin								Region of origin of migrant cases															
	Total		Non-migrants		Migrants born in the EU/EEA		Migrants born outside of the EU/EEA		Western Europe		Central Europe		Eastern Europe		Sub-Saharan Africa		Latin America and Caribbean		South and South-east Asia		Other		Unknown	
Number	103,416		55,943		6,337		41,136		3,460		4,707		5,335		21,232		6,601		3,119		3,019		19,910	
Gender																								
Women	25,751	24.9	7,751	13.9	1,026	16.2	16,974	41.3	391	11.3	812	17.3	2,176	40.8	12,230	57.6	1,095	16.6	833	26.7	463	15.3	6,838	34.3
Men	76,921	74.4	48,023	85.8	5,280	83.3	23,618	57.4	3,058	88.4	3,863	82.1	3,140	58.9	8,940	42.1	5,117	77.5	2,250	72.1	2,530	83.8	13,012	65.4
Transgender	713	0.7	164	0.3	31	0.5	518	1.3	11	0.3	32	0.7	14	0.3	50	0.2	383	5.8	34	1.1	25	0.8	25	0.1
Unknown	31	0	5	0	0	0	26	0.1	0	0	0	0	5	0.1	12	0.1	6	0.1	2	0.1	1	0	35	0.2
Male:female ratio^a^	3		6.2		5.1		1.4		7.8		4.8		1.4		0.7		4.7		2.7		5.5	1.9		
Median age in years (IQR)	37 (29–47)		39 (30–50)		35 (28–44)		35 (28–43)		39 (30–48)		34 (28–42)		38 (32–44)		35 (28–43)		32 (27–39)		34 (28–42)		35 (28–44)		39 (30–49)	
Age group in years																								
≤ 18	1,797	1.7	617	1.1	59	0.9	1,121	2.7	23	0.7	42	0.9	140	2.6	824	3.9	52	0.8	38	1.2	61	2	444	2.2
19–29	26,278	25.4	13,286	23.7	1,784	28.2	11,208	27.2	819	23.7	1,421	30.2	837	15.7	5,521	26	2,588	39.2	925	29.7	881	29.2	3,992	20.1
30–50	56,299	54.4	28,691	51.3	3,710	58.5	23,898	58.1	1,933	55.9	2,762	58.7	3,782	70.9	12,166	57.3	3,445	52.2	1,859	59.6	1,661	55	11,070	55.6
> 50	18,929	18.3	13,286	23.7	774	12.2	4,869	11.8	679	19.6	473	10	567	0.6	2,709	12.8	512	7.8	290	9.3	413	13.7	4,324	21.7
Unknown	113	0.1	63	0.1	10	0.2	40	0.1	6	0.2	9	0.2	9	0.2	12	0.1	4	0.1	7	0.2	3	0.1	80	0.4
Mode of transmission																								
Sex between men	45,739	44.2	31,867	57	3,348	52.8	10,524	25.6	2,168	62.7	2,234	47.5	844	15.8	1,727	8.1	4,042	61.2	1,456	46.7	1,401	46.4	1,777	8.9
Heterosexual transmission (men)	17,606	17	8,239	14.7	736	11.6	8,631	21	444	12.8	558	11.9	804	15.1	5,959	28.1	759	11.5	283	9.1	560	18.5	918	4.6
Heterosexual transmission (women)	21,483	20.8	5,974	10.7	745	11.8	14,764	35.9	288	8.3	591	12.6	1,631	30.6	10,951	51.6	987	15	713	22.9	348	11.5	1,145	5.8
Injecting drug use	4,588	4.4	2,776	5	509	8	1,303	3.2	134	3.9	356	7.6	985	18.5	67	0.3	38	0.6	131	4.2	101	3.3	460	2.3
Mother to child transmission	615	0.6	141	0.3	27	0.4	447	1.1	14	0.4	12	0.3	105	2	294	1.4	9	0.1	17	0.5	23	0.8	42	0.2
Other routes	217	0.2	34	0.1	15	0.2	168	0.4	6	0.2	13	0.3	29	0.5	106	0.5	6	0.1	13	0.4	10	0.3	5	0
Unknown	13,168	12.7	6,912	12.4	957	15.1	5,299	12.9	406	11.7	943	20	937	17.6	2,128	10	760	11.5	506	16.2	576	19.1	15,563	78.2
Median CD4^+^ T-cell count/μL (IQR)	350 (164–544)		372 (176–564)		378 (183–579)		316 (150–507)		386 (204–589)		360 (147–558)		355 (126–581)		294 (143–473)		363 (199–546)		268 (93–454)		357 (175–544)		310 (118–530)	
CD4^+^ category at HIV diagnosis																								
< 200 CD4^+^ T-cell count/μL	21,073	20.4	10,770	19.3	1,134	17.9	9,169	22.3	600	17.3	917	19.5	938	17.6	5,205	24.5	1,251	19	781	25	611	20.2	835	4.2
200 to < 350 CD4^+^ T-cell count/μL	14,909	14.4	7,558	13.5	808	12.8	6,543	15.9	491	14.2	545	11.6	520	9.7	3,742	17.6	1,128	17.1	467	15	458	15.2	471	2.4
350 to < 500 CD4^+^ T-cell count/μL	14,590	14.1	8,252	14.8	868	13.7	5,470	13.3	496	14.3	599	12.7	492	9.2	2,882	13.6	1,082	16.4	344	11	443	14.7	412	2.1
≥ 500 CD4^+^ T-cell count/μL	21,639	20.9	12,792	22.9	1,430	22.6	7,417	18	883	25.5	957	20.3	990	18.6	3,444	16.2	1,510	22.9	398	12.8	665	22	672	3.4
Unknown	31,205	30.2	16,571	29.6	2,097	33.1	12,537	30.5	990	28.6	1,689	35.9	2,395	44.9	5,959	28.1	1,630	24.7	1,129	36.2	842	27.9	17,520	88
AIDS at HIV diagnosis	14,092	13.6	7,893	14.1	843	13.3	5,356	13	405	11.7	704	15	766	14.4	2,762	13	649	9.8	536	17.2	377	12.5	679	3.4
Reporting EU/EEA subregion																								
Eastern EU/EEA subregion	5,930	5.7	4,951	8.9	240	3.8	739	1.8	35	1	198	4.2	618	11.6	20	0.1	37	0.6	50	1.6	21	0.7	1,926	9.7
Southern EU/EEA subregion	17,614	17	11,002	19.7	391	6.2	6,221	15.1	451	13	583	12.4	509	9.5	3,031	14.3	1,617	24.5	266	8.5	155	5.1	1,318	6.6
Western EU/EEA suubregion	72,220	69.8	37,092	66.3	5,133	81	29,995	72.9	2,621	75.8	3,547	75.4	3,746	70.2	16,223	76.4	4,319	65.4	2,114	67.8	2,558	84.7	15,739	79.1
Northern EU/EEA subregion	7,652	7.4	2,898	5.2	573	9	4,181	10.2	353	10.2	379	8.1	462	8.7	1,958	9.2	628	9.5	689	22.1	285	9.4	927	4.7

EEA: European Economic Area; EU: European Union; IQR: interquartile range.

^a^ Male-to-female ratio indicates the proportion of male to female in the population, expressed as a ratio.

For this analysis, the primary exposure variable, geographical origin, was determined using information on country of birth, nationality and/or region of origin. When multiple variables were available, priority was given in the following order: country of birth, nationality and then region of origin. Geographical origin was classified as non-migrant if the reporting country matched the country of birth or nationality. The non-migrant population was defined as people whose reported place of birth was the reporting country. Two regional categories were established: ‘region of origin’, based on UNAIDS designation and divided into non-EU/EEA and EU/EEA countries to define migrants' birth regions, and ‘EU/EEA subregions' representing reporting countries within the EU/EEA as listed in the appended Supplementary Tables S1 and S2. Countries excluded from the analysis were: Bulgaria, Croatia, Finland, Hungary, Italy, Liechtenstein, Lithuania, Malta, Poland, Romania, Slovenia and Spain because they were unable to classify diagnoses as either new or previously positive.

For those where the respective information was known, migrants born in EU/EEA countries were predominantly men (83.3%; n = 5,280), aged 30–50 years (58.5%: n = 3,710) and with sex between men as the primary mode of transmission (52.8%; n = 3,338). Among migrants from non-EU/EEA countries there was a higher proportion of women (41.3%; n = 16,974), with most aged 30–50 years (58.1%; n = 23,898) and heterosexual sex was the most common mode of transmission (56.9%; n = 23,395) (Table 1). Overall, migrant women tended to be slightly younger than migrant men and were most likely to acquire HIV through heterosexual transmission (83.4%), while sex between men remains the leading mode of transmission for men (59.3%). We conducted a descriptive analysis by gender, which is appended in Supplementary Table S3.

## HIV diagnosis trends among migrant populations in EU/EEA countries

The HIV diagnosis trends among migrant populations are presented in the Figure. For purposes of comparison, we also present data for non-migrants. Between 2014 and 2023, HIV diagnoses reported by EU/EEA countries in migrants originating from non-EU/EEA countries increased by 14.4%, while diagnoses in people originating from another EU/EEA country decreased by 24.6%. The increase in new HIV diagnoses among migrants from non-EU/EEA countries was greater in men (16.7%) than in women (8.1%) (Figure, panel A). The largest rise in reporting of new HIV diagnoses among migrants was seen from 2021 to 2023 in the western EU/EEA subregion (32.1%; from 6,456 to 8,531 diagnoses) followed by the northern (29.0%, from 610 to 787) and eastern (7.5%, from 591 to 635 diagnoses) subregions (Figure, panel B). Since 2014, reported HIV diagnoses among migrants from different regions of origin have shown distinct trends (Figure, panel C). In men from Sub-Saharan Africa, there was a steady decline from 2014 to 2019, a sharp drop in 2020, probably due to the COVID-19 pandemic, followed by an increase in 2021 (Figure, panel D). The pattern in women was similar but with a sharper increase beginning in 2022, rising by 59.2% from 2021 to 2023 (Figure, panel E). For people from eastern Europe, diagnoses rose in 2022 for both men and women, probably due to the conflict in Ukraine [2]. Diagnoses in men from Latin America and the Caribbean increased from 2014 to 2019, declined until 2021, followed by an increase by 43.8% through 2023 (Figure, panel D).

**Figure:**
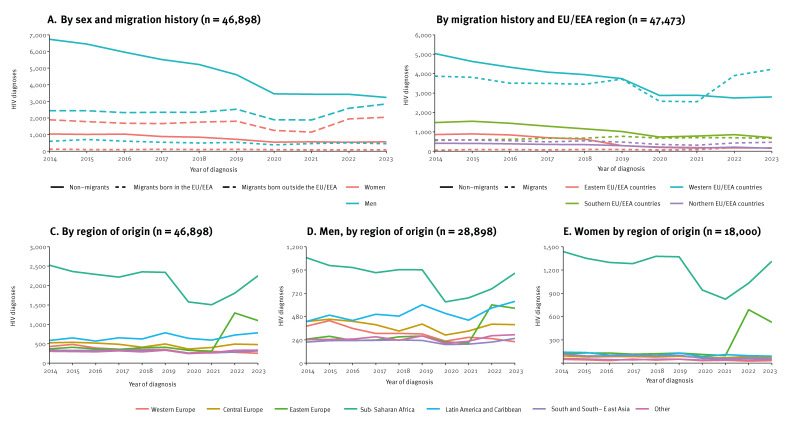
HIV diagnosis trends among migrant and non-migrant populations, EU/EEA, 2014–2023

EEA: European Economic Area; EU: European Union.

Geographical origin was classified as non-migrant if the reporting country matched the country of birth or nationality. The non-migrant population was defined as people whose reported place of birth was the reporting country. Two regional categories were established: ‘region of origin’, based on UNAIDS designation and divided into non-EU/EEA and EU/EEA countries to define migrants' birth regions, and ‘EU/EEA subregions' representing reporting countries within the EU/EEA as listed in the appended Supplementary Tables S1 and S2. Countries excluded from the analysis were: Bulgaria, Croatia, Finland, Hungary, Italy, Liechtenstein, Lithuania, Malta, Poland, Romania, Slovenia and Spain because they were unable to classify diagnoses as either new or previously positive.

## Late diagnosis

Late diagnosis is defined as an HIV diagnosis with a CD4^+^ T-cell count below 350 cells/μL or an AIDS-defining event; cases diagnosed during the acute stage are not classified as late, even when the individual has a low CD4^+^ T-cell count [3]. The percentage of late HIV diagnoses was 52.4% among all migrants, 43.1% in EU/EEA-born migrants and 53.8% in non-EU/EEA migrants. In comparison, the percentage of late HIV diagnoses among non-migrants was 42.6%. The EU/EEA-born migrants diagnosed late were predominantly men (82.6%; n = 1,394). The main transmission mode in this group was sex between men (50.5%; n = 852). Late diagnoses in EU/EEA-born migrants were reported primarily by western EU/EEA countries (72.8%; n = 1,229). For non-EU/EEA-born migrants, late diagnoses were more balanced between men (56.2%; n = 7,944) and women (43.8%; n = 6,182), with a median age of 37 years (range: 30–45). Heterosexual transmission was the dominant mode of transmission, responsible for 54.4% (n = 10,355) of those diagnosed late. Late diagnoses among non-EU/EEA-born cases were also mostly reported by western EU/EEA countries (69.3%; n = 9,788) (Table 2).

**Table 2:** Sociodemographic characteristics of migrant and non-migrant populations with late and non-late HIV diagnoses and mode of transmission, EU/EEA, 2014–2023 (n = 67,677)

	Non-late diagnosis among non-migrants		Late diagnosis among non-migrants		Non-late diagnosis among migrants born in EU/EEA		Late diagnosis among migrants born in EU/EEA		Non-late diagnosis among migrants born outside the EU/EEA		Late diagnosis among migrants born outside the EU/EEA	
Number	21,510		15,985		2,225		1,688		12,143		14,126	
Gender												
Women	2,720	12.6	2,340	14.6	256	11.5	294	17.4	4,754	39.2	6,182	43.8
Men	18,790	87.4	13,645	85.4	1,969	88.5	1,394	82.6	7,389	60.8	7,944	56.2
Male:female ratio^a^	6.9		5.8		7.7		4.7		1.6		1.3	
Median age in years (IQR)	35 (27–46)		44 (34–54)		33 (27–41)		38 (31–47)		33 (27–41)		37 (30–45)	
Age group in years												
≤ 18	281	1.3	50	0.3	22	1.0	5	0.3	237	2.0	188	1.3
19–29	6,908	32.1	2,321	14.5	768	34.5	346	20.5	4,074	33.6	3,085	21.8
30–50	10,862	50.5	8,414	52.6	1,235	55.5	1,055	62.5	6,679	55.0	8,742	61.9
> 50	3,459	16.1	5,200	32.5	200	9.0	282	16.7	1,153	9.5	2,111	14.9
Mode of transmission												
Sex between men	15,299	71.1	8,770	54.9	1,615	72.6	852	50.5	4,718	38.9	3,177	22.5
Heterosexual transmission (men)	2,817	13.1	4,146	25.9	236	10.6	392	23.2	2,388	19.7	4,315	30.5
Heterosexual transmission (women)	2,467	11.5	2,165	13.5	228	10.2	274	16.2	4,641	38.2	6,040	42.8
Injecting drug use	911	4.2	883	5.5	142	6.4	157	9.3	306	2.5	454	3.2
Mother to child transmission	3	0.0	5	0.0	2	0.1	3	0.2	42	0.3	53	0.4
Other routes	13	0.1	16	0.1	2	0.1	10	0.6	48	0.4	87	0.6
Median CD4^+^ T-cell count/μL (IQR)	524 (414–687)		160 (55–264)		540 (423–703)		175 (57–277)		511 (409–659)		171 (65–267)	
CD4^+^ T-cell count category at HIV diagnosis												
< 200 CD4^+^ T-cell count/μL^b^	556	2.6	8,727	54.6	54	2.4	889	52.7	320	2.6	7,678	54.4
200 to < 350 CD4^+^ T-cell count/μL^b^	1,370	6.4	5,660	35.4	117	5.3	627	37.1	608	5.0	5,340	37.8
350 to < 500 CD4^+^ T-cell count/μL	7,631	35.5	195	1.2	764	34.3	36	2.1	4,784	39.4	206	1.5
≥ 500 CD4^+^ T-cell count/μL	11,927	55.4	283	1.8	1,282	57.6	46	2.7	6,423	52.9	195	1.4
Unknown	26	0.1	1,120	7.0	8	0.4	90	5.3	8	0.1	707	5.0
AIDS at HIV diagnosis	132	0.6	6,160	38.5	17	0.8	625	37.0	58	0.5	4,489	31.8
Reporting EU/EEA region												
Eastern EU/EEA countries	1,612	7.5	1,444	9.0	128	5.8	65	3.9	202	1.7	245	1.7
Southern EU/EEA countries	4,265	19.8	4,776	29.9	139	6.2	197	11.7	1,982	16.3	2,724	19.3
Western EU/EEA countries	14,602	67.9	8,829	55.2	1,749	78.6	1,229	72.8	8,941	73.6	9,788	69.3
Northern EU/EEA countries	1,031	4.8	936	5.9	209	9.4	197	11.7	1,018	8.4	1,369	9.7

EEA: European Economic Area; EU: European Union; IQR: interquartile range.

^a^ Male-to-female ratio indicates the proportion of male to female in the population, expressed as a ratio.

^b^ CD4^+^ T-cell cell count < 350 cells/μL among people with non-late diagnoses is likely to indicate diagnosis during the acute phase of HIV infection. Acute HIV infection was defined as a primary HIV infection in the initial stage following HIV acquisition, marked by high levels of HIV RNA or p24 antigen in the blood before detectable antibodies developed. This phase typically occurs 2–4 weeks after exposure and may present with low CD4^+^ T-cell cell count [3]. Geographical origin was classified as non-migrant if the reporting country matched the country of birth or nationality. The non-migrant population was defined as people whose reported place of birth was the reporting country. Two regional categories were established: ‘region of origin’, based on UNAIDS designation and divided into non-EU/EEA and EU/EEA countries to define migrants' birth regions, and ‘EU/EEA subregions' representing reporting countries within the EU/EEA as listed in the appended Supplementary Tables S1 and S2. Countries excluded from the analyses were: Bulgaria, Croatia, Finland, Hungary, Italy, Liechtenstein, Lithuania, Malta, Poland, Romania, Slovenia and Spain because they were unable to classify diagnoses as either new or previously positive.

Cases with gender marked as Transgender or Unknown, cases lacking age information, cases with age ≤ 15 years, cases with unknown migration status or mode of transmission and cases without AIDS diagnosis and unknown CD4^+^ T-cell count were excluded from this analysis.

Table 3 describes the results of a modified Poisson model [4] used to assess risk factors for late HIV diagnosis, stratified by migration from EU/EEA and non-EU/EEA countries and by sex. Predictors included age, transmission mode, reporting country and year of diagnosis (pre-COVID-19 (2014–2019) vs COVID-19/post-COVID-19 (2020–2023). Age analysis indicated that the ratio increased with age among migrant men from both EU/EEA and non-EU/EEA countries, and among women from non-EU/EEA countries. The prevalence ratio (PR) was highest among men older than 50 years born in EU/EEA countries (PR = 2.89; 95% confidence interval (CI): 1.25–6.69) and non-EU/EEA countries (PR = 1.39; 95% CI: 1.18–1.64), as well as among women older 50 years born in non-EU/EEA countries (PR = 1.44; 95% CI: 1.22–1.69), compared with people aged 18 years or younger. Regionally, male migrants born in the EU/EEA diagnosed with HIV in the southern EU/EEA subregion had a significantly higher PR of late diagnosis than those in the northern subregion, while those in the western and eastern EU/EEA subregions had a significantly lower PR compared with the northern subregion (Table 3). Migrant men and women from the EU/EEA had a 6% (PR = 1.06; 95% CI: 1.02–1.10) and 25% (PR = 1.25; 95% CI: 1.15–1.36) higher PR, respectively, of late HIV diagnosis than non-migrants. This ratio was even higher for people born outside the EU/EEA, with a PR of 19% in men (PR = 1.19; 95% CI: 1.17–1.22) and 31% in women (PR = 1.31; 95% CI: 1.26–1.36) compared with non-migrants. We applied an extra-Poisson model stratified by sex to assess the PR of late diagnosis among migrants compared to the non-migrant population, as shown in Supplementary Table S4.

**Table 3:** Risk factors for late HIV diagnosis among EU/EEA and non-EU/EEA migrants: modified Poisson model, EU/EEA, 2014–2023 (n = 30,182)

	Men				Women			
	Migrants born outside of the EU/EEA		Migrants born in the EU/EEA		Migrants born outside of the EU/EEA		Migrants born in the EU/EEA	
	PR	95% CI	PR	95% CI	PR	95% CI	PR	95% CI
Time period of diagnosis^a^								
Pre-COVID	Reference							
Post-COVID	1.00	0.97–1.03	1.03	0.95–1.11	0.98	0.94–1.01	0.88	0.75–1.05
Age group in years								
≤ 18	Reference							
19–29	1.02	0.86–1.20	1.67	0.72–3.87	1.12	0.96–1.31	3.09	0.53–18.12
30–50	1.25	1.06–1.47	2.31	1.00–5.34	1.37	1.17–1.60	4.25	0.73–24.75
> 50	1.39	1.18–1.64	2.89	1.25–6.69	1.44	1.22–1.69	4.54	0.78–26.56
Reporting EU/EEA region								
Northern EU/EEA countries	Reference							
Western EU/EEA countries	0.89	0.85–0.94	0.86	0.77–0.97	0.88	0.83–1.92	0.84	0.67–1.06
Eastern EU/EEA countries	0.87	0.77–0.98	0.73	0.58–0.92	0.98	0.86–1.11	0.81	0.45–1.45
Southern EU/EEA countries	1.00	0.95–1.06	1.22	1.05–1.41	0.96	0.90–1.02	1.02	0.76–1.36
Mode of transmission								
Heterosexual transmission	Reference							
Injecting drug use	0.93	0.87–0.99	0.91	0.80–1.04	0.96	0.81–1.14	0.78	0.56–1.09
Sex between men	0.66	0.64–0.69	0.61	0.56–0.67	NA			
Mother to child transmission	1.29	1.02–1.62	2.74	2.01–3.74	1.02	0.74–1.40	ND	
Other routes	1.17	0.97–1.41	1.27	0.93–1.71	1.05	0.89–1.23	ND	

CI: confidence interval; EEA: European Economic Area; EU: European Union; NA: not applicable; ND: not determined due to low number of reported HIV diagnoses; PR: prevalence ratio.

^a^ The years 2014–2019 are here defined as pre-COVID-19, the years 2020–2023 as post-COVID-19.

For each stratum, the model estimates report the PR, obtained by exponentiating the β coefficients from the robust Poisson model estimation, along with the corresponding 95% CIs. Countries excluded from the analyses were: Bulgaria, Croatia, Finland, Hungary, Italy, Liechtenstein, Lithuania, Malta, Poland, Romania, Slovenia and Spain because were unable to classify diagnoses as either new or previously positive.

## Discussion

Nearly half of all new HIV diagnoses reported by EU/EEA countries between 2014 and 2023 were among migrants, and this proportion increased over time. Migration flows within the EU/EEA have remained broadly stable at around 2 million per year during the past 10 years. Migration from non-EU countries has fluctuated but increased overall during the same time period [5]. Most migrants diagnosed with HIV in our study originated from non-EU/EEA countries, particularly Sub-Saharan Africa, Latin America and the Caribbean, and eastern Europe. Migrants from other EU/EEA countries were predominantly men, with HIV primarily transmitted through sex between men, whereas migrants from non-EU/EEA countries included a higher proportion of women, with heterosexual transmission as the most common mode of transmission.

While this study lacked data on whether migrants acquired HIV before or after migration, evidence suggests that many migrants, including those from high-prevalence regions, contract HIV after arriving to the EU/EEA [6,7]. An elevated risk of HIV acquisition among migrants, particularly men who have sex with men (MSM), has been described and is likely to reflect increased vulnerability after migration [8]. This highlights the need for comprehensive sexual health services, including condom distribution and access to pre-exposure prophylaxis (PrEP) for high-risk, HIV-negative MSM and migrant populations at high risk for HIV [9]. Enhancing surveillance to accurately classify migrants and record arrival dates is essential for determining whether HIV acquisition occurs before or after migration. Countries in the EU/EEA should adopt evidence-based, long-term prevention strategies that include structural, behavioural and biomedical interventions tailored to high-prevalence migrant groups and MSM.

Late HIV diagnosis is an important and escalating concern in the EU/EEA region which reached a peak of 52.7% in 2023 [1]. Our findings describe high rates of late HIV diagnosis in both migrant and non-migrant populations. Among migrants, the risk was higher in non-EU/EEA men and women older than 50 years, primarily infected through heterosexual transmission, and late diagnoses tended to be reported more frequently in the southern EU/EEA subregion. Late diagnosis leads to higher morbidity and mortality and increases the likelihood of onward HIV transmission [10]. Migrants from high-prevalence countries often arrive in host countries with advanced HIV infection and thus preventing advanced HIV infection by early diagnosis is challenging [11]. However, increased awareness among healthcare providers is vital, as late diagnosis occurs more commonly in heterosexual migrants, possibly related to misconceptions about HIV risk in heterosexuals [12].

Given the high and increasing proportion of HIV diagnoses and late diagnosis among migrants in the last year, it is essential to develop, implement and expand migrant-targeted strategies that enhance access to HIV testing and linkage to care in host countries. Barriers to HIV testing for this group include limited healthcare access, insufficient information on available services, low perceived HIV risk, unclear policies on HIV and sexual transmitted infections testing at sexual health centres and missed testing opportunities in general practice [12]. According to monitoring data on the HIV response, self-testing and community-based testing are still not universally provided to migrant populations across the EU/EEA [13] and need to be scaled up. To effectively reach this group, prevention programmes should prioritise regular, accessible HIV testing with immediate linkage to care. Scaling up testing for indicator conditions, testing in emergency department, community-based testing, reminders for clinicians, peer support to help migrants navigate the health system, and self-testing options may enhance testing uptake among migrants [14]. Several EU/EEA countries report implementation of migrant-sensitive approaches. Sharing these experiences may support countries facing similar issues.

Incorporating HIV prevention and treatment into a broader health delivery approach can reduce issues of stigma as well as financial barriers for migrants. Integrating links between HIV support and other services such as social services is often necessary to address patient needs and is particularly important for undocumented migrants where barriers to accessing services may be substantial in some EU/EEA countries [13]. To effectively reach migrant populations, inclusive research and service design of community-based, culture- and language-tailored efforts including peer-to-peer involvement are essential to increase uptake of services.

Our analysis has several limitations. Firstly, the absence of data on the time from migration to diagnosis restricts our understanding of missed opportunities for earlier HIV diagnosis in the EU/EEA. In addition, late diagnosis rates may be slightly overestimated if acute infections are misclassified as delayed diagnoses rather than recent infections. Also, relevant changes in HIV reporting systems during the study period have not been taken in consideration and might affect the results of this analysis, leading to possible biased trends in the number of HIV-positive migrants reported. It is important to note that the analysis excluded previous positive diagnoses, as this falls outside the scope of this analysis. The focus was specifically on new diagnoses to provide evidence for shaping targeted testing policies. Lastly, as 12 EU/EEA countries contributing to HIV surveillance were not included in the analysis, the presented results might not reflect the situation in the whole EU/EEA. Enhancing surveillance by incorporating the diagnosis status variable would improve characterisation of new diagnoses across countries.

## Conclusion

Migrant populations in the EU/EEA are diverse and are disproportionately affected by HIV. Late diagnosis in migrant populations is high generally and particularly high in some migrant sub-groups. Enhanced efforts are required to effectively address HIV prevention and testing needs in the diverse population of migrants who are at risk for or living with HIV in EU/EEA countries.

## Note

The views and opinions expressed in this paper are those of the authors and not necessarily the views and decisions or policies of the European Centre for Disease Prevention and Control.

## Supplementary Data

412000 Supplement
