# Authors’ correction for Euro Surveill. 2024;29(48)

**DOI:** 10.2807/1560-7917.ES.2024.29.49.241205c

**Published:** 2024-12-05

**Authors:** Eurosurveillance editorial team

**Affiliations:** 1 European Centre for Disease Prevention and Control (ECDC), Stockholm, Sweden

In the article entitled ‘Trends in new HIV diagnoses and factors contributing to late diagnosis among migrant populations in EU/EEA countries, 2014 to 2023’ by Reyes-Urueña et al., published on 28 November 2024, the authors Ivailo Alexiev and Ard van Sighem had been omitted from the list of collaborators. The names were added at the request of the corresponding author on 3 December 2024 and 5 December 2024, respectively. 

