# Supplementary material for: Trends in new HIV diagnoses and factors contributing to late diagnosis among migrant populations in EU/EEA countries, 2014 to 2023
Source: Euro Surveill. 2024 Nov 28;29(48):2400759. doi: 10.2807/1560-7917.ES.2024.29.48.2400759 (PMC11605804; doi:10.2807/1560-7917.ES.2024.29.48.2400759)
Supplement: Supplement [file 24-00759_REYES_Supplement.pdf]

This supplementary material is hosted by *Eurosurveillance* as supporting information alongside the article [Trends in new HIV diagnoses and factors contributing to late diagnosis among migrant populations in EU/EEA countries, 2014 to 2023], on behalf of the authors, who remain responsible for the accuracy and appropriateness of the content. The same standards for ethics, copyright, attributions and permissions as for the article apply. Supplements are not edited by *Eurosurveillance* and the journal is not responsible for the maintenance of any links or email addresses provided therein.

Table S1. Country distribution by reporting subregion

Reporting subregions			
**Eastern EU/EEA subregion**	**Southern EU/EEA subregion**	**Western EU/EEA subregion**	**Northern EU/EEA subregion**
*Bulgaria (BG)<br>Czech Republic (CZ)<br>*Hungary (HU)<br>*Poland (PL)<br>*Romania (RO)<br>Slovakia (SK)<br>Estonia (EE)<br>Latvia (LV)<br>*Lithuania (LT)	*Croatia (HR)<br>Cyprus (CY)<br>Greece (EL)<br>*Italy (IT)<br>*Malta (MT)<br>Portugal (PT)<br>*Slovenia (SI)<br>*Spain (ES)	Austria (AT)<br>Belgium (BE)<br>France (FR)<br>Germany (DE)<br>Liechtenstein (LI)<br>Luxembourg (LU)<br>Netherlands (NL)	Denmark (DK)<br>*Finland (FI)<br>Iceland (IS)<br>Ireland (IE)<br>Norway (NO)<br>Sweden (SE)

*These countries were excluded from the analyses because they were unable to classify diagnoses as either new or previously positive.

Table S2. Country distribution by region of origin based on UNAIDS designation

Geographical region of origin			
**Western Europe**	**Central Europe**	**Eastern Europe**	**Latin America and Caribbean**
Andorra (AD)<br>Austria (AT)<br>Belgium (BE)<br>Denmark (DK)<br>Finland (FI)<br>Faroe Islands (FO)<br>France (FR)<br>Germany (DE)<br>Greenland (GL)<br>Greece (EL)<br>Iceland (IS)<br>Ireland (IE)<br>Israel (IL)<br>Italy (IT)<br>Liechtenstein (LI)<br>Luxembourg (LU)<br>Malta (MT)<br>Monaco (MC)<br>Netherlands (NL)<br>Norway (NO)<br>Portugal (PT)<br>San Marino (SM)<br>Spain (ES)<br>Sweden (SE)<br>Switzerland (CH)<br>United Kingdom (UK)	Bosnia and Herzegovina (BA)<br>Bulgaria (BG)<br>Croatia (HR)<br>Cyprus (CY)<br>Czech Republic (CZ)<br>Hungary (HU)<br>North Macedonia (MK)<br>Poland (PL)<br>Romania (RO)<br>Serbia and Montenegro (CS) *(no longer in use)*<br>Slovakia (SK)<br>Slovenia (SI)<br>Turkey (TR)<br>Yugoslavia (YU) *(no longer in use)*<br>Kosovo (XK)<br>Serbia (RS)	Armenia (AM)<br>Azerbaijan (AZ)<br>Belarus (BY)<br>Estonia (EE)<br>Georgia (GE)<br>Kazakhstan (KZ)<br>Kyrgyzstan (KG)<br>Latvia (LV)<br>Lithuania (LT)<br>Montenegro (ME)<br>Moldova (MD)<br>Russia (RU)<br>Tajikistan (TJ)<br>Turkmenistan (TM)<br>Ukraine (UA)<br>Uzbekistan (UZ)	Antigua and Barbuda (AG)<br>Netherlands Antilles (AN)<br>Argentina (AR)<br>Uruguay (UY)<br>Bahamas (BS)<br>Barbados (BB)<br>Cuba (CU)<br>Dominica (DM)<br>Dominican Republic (DO)<br>Grenada (GD)<br>Guadeloupe (GP)<br>Haiti (HT)<br>Jamaica (JM)<br>Saint Kitts and Nevis (KN)<br>Saint Lucia (LC)<br>Montserrat (MS)<br>Martinique (MQ)<br>Trinidad and Tobago (TT)<br>Saint Vincent and the Grenadines (VC)<br>Belize (BZ)<br>Bolivia (BO)<br>Brazil (BR)<br>Chile (CL)<br>Colombia (CO)<br>Costa Rica (CR)<br>Ecuador (EC)<br>El Salvador (SV)<br>French Guiana (GF)<br>Guatemala (GT)<br>Guyana (GY)<br>Honduras (HN)<br>Mexico (MX)<br>Nicaragua (NI)<br>Panama (PA)<br>Paraguay (PY)<br>Peru (PE)<br>Puerto Rico (PR)<br>Turks and Caicos (TC)

			Venezuela (VE)<br>Suriname (SR)

**Geographical region of origin**			
**Sub Saharan Africa**		**South and Southeast Asia**	**Other**
Angola (AO)<br>Benin (BJ)<br>Botswana (BW)<br>Burkina Faso (BF)<br>Burundi (BI)<br>Cameroon (CM)<br>Central African Republic (CF)<br>Cape Verde (CV)<br>Comoros (KM)<br>Republic of the Congo (CG)<br>Democratic Republic of the Congo (CD)<br>Ivory Coast (CI)<br>Djibouti (DJ)<br>Equatorial Guinea (GQ)<br>Eritrea (ER)<br>Ethiopia (ET)<br>Gabon (GA)<br>Gambia (GM)<br>Ghana (GH)<br>Guinea (GN)<br>GuineaBissau (GW)<br>Kenya (KE)	Lesotho (LS)<br>Liberia (LR)<br>Madagascar (MG)<br>Malawi (MW)<br>Mali (ML)<br>Mauritania (MR)<br>Mauritius (MU)<br>Mozambique (MZ)<br>Namibia (NA)<br>Namibia (NAM)<br>*(alternative code for Namibia)*<br>Nigeria (NG)<br>Rwanda (RW)<br>Senegal (SN)<br>Sierra Leone (SL)<br>Somalia (SO)<br>South Africa (ZA)<br>Eswatini (SZ)<br>Tanzania (TZ)<br>Togo (TG)<br>Uganda (UG)<br>Zambia (ZM)<br>Zimbabwe (ZW)<br>Mayotte (YT)<br>Seychelles (SC)<br>Chad (TD)<br>Niger (NE)<br>South Sudan (SS)	China (CN)<br>Macau (MO)<br>Fiji (FJ)<br>Japan (JP)<br>North Korea (KP)<br>South Korea (KR)<br>Mongolia (MN)<br>Papua New Guinea (PG)<br>Solomon Islands (SB)<br>Tonga (TO)<br>Taiwan (TW)<br>Bangladesh (BD)<br>Bhutan (BT)<br>Brunei (BN)<br>Cambodia (KH)<br>Hong Kong (HK)<br>India (IN)<br>Indonesia (ID)<br>Iran (IR)<br>Laos (LA)<br>Malaysia (MY)<br>Maldives (MV)<br>Myanmar (MM)<br>Nepal (NP)<br>Pakistan (PK)<br>Philippines (PH)<br>Singapore (SG)<br>Sri Lanka (LK)<br>Thailand (TH)<br>Vietnam (VN)<br>TimorLeste (TL)<br>Afghanistan (AF)	Algeria (DZ)<br>Bahrain (BH)<br>Egypt (EG)<br>Iraq (IQ)<br>Jordan (JO)<br>Kuwait (KW)<br>Lebanon (LB)<br>Libya (LY)<br>Morocco (MA)<br>Oman (OM)<br>Qatar (QA)<br>Saudi Arabia (SA)<br>Sudan (SD)<br>Soviet Union (SU)<br>*(no longer in use)*<br>Syria (SY)<br>Tunisia (TN)<br>United Arab Emirates (AE)<br>Yemen (YE)<br>United States (US)<br>Christmas Island (CX)<br>New Zealand (NZ)<br>Canada (CA)<br>Australia (AU)<br>Palestine (PS)

Classified as ECDC NORMAL

Table S3. Sociodemographic characteristics of migrant and non-migrant populations diagnosed with HIV in EU/EEA countries by sex (2014–2023), n=123,326.

Classified as ECDC NORMAL

Variable	Total	Non-migrants	Migrants born in the EU/EEA	Migrants born out the EU/EEA	Western Europe	Central Europe	Eastern Europe	Sub-Saharan Africa	Latin America and Caribbean	South and South-east Asia	Other	Unknown
**Women**	25,751	7,751	1,026	16,974	391	812	2,176	12,230	1,095	833	463	6,838
**Median age, years (IQR)**	36 (29, 45)	39 (29, 51)	34 (27, 43)	35 (29, 43)	39 (29, 50)	34 (27, 43)	38 (32, 45)	34 (28, 42)	37 (30, 45)	39 (33, 45)	35 (27, 46)	38 (30, 48)
**Age category**												
≤18	751 (2.9%)	195 (2.5%)	20 (1.9%)	536 (3.2%)	6 (1.5%)	17 (2.1%)	64 (2.9%)	413 (3.4%)	27 (2.5%)	12 (1.4%)	17 (3.7%)	212 (3.1%)
19-29	6,289 (24.4%)	1,785 (23.0%)	323 (31.5%)	4,181 (24.6%)	94 (24.0%)	260 (32.0%)	314 (14.4%)	3,358 (27.5%)	235 (21.5%)	108 (13.0%)	135 (29.2%)	1,351 (19.8%)
30-50	14,476 (56.2%)	3,681 (47.5%)	543 (52.9%)	10,252 (60.4%)	198 (50.6%)	438 (53.9%)	1,518 (69.8%)	7,140 (58.4%)	659 (60.2%)	610 (73.2%)	232 (50.1%)	3,946 (57.7%)
>50	4,219 (16.4%)	2,084 (26.9%)	137 (13.4%)	1,998 (11.8%)	91 (23.3%)	96 (11.8%)	276 (12.7%)	1,317 (10.8%)	173 (15.8%)	103 (12.4%)	79 (17.1%)	1,314 (19.2%)
Unknown	16 (0.1%)	6 (0.1%)	3 (0.3%)	7 (0.0%)	2 (0.5%)	1 (0.1%)	4 (0.2%)	2 (0.0%)	1 (0.1%)	0 (0.0%)	0 (0.0%)	15 (0.2%)
**Mode of transmission**												
Heterosexual transmission (women)	21,483 (83.4%)	5,974 (77.1%)	745 (72.6%)	14,764 (87.0%)	288 (73.7%)	591 (72.8%)	1,631 (75.0%)	10,951 (89.5%)	987 (90.1%)	713 (85.6%)	348 (75.2%)	1,145 (16.7%)
Injecting drug use	933 (3.6%)	650 (8.4%)	78 (7.6%)	205 (1.2%)	28 (7.2%)	43 (5.3%)	180 (8.3%)	17 (0.1%)	3 (0.3%)	5 (0.6%)	7 (1.5%)	78 (1.1%)
Mother to child transmission	313 (1.2%)	70 (0.9%)	13 (1.3%)	230 (1.4%)	7 (1.8%)	8 (1.0%)	50 (2.3%)	149 (1.2%)	7 (0.6%)	10 (1.2%)	12 (2.6%)	23 (0.3%)
Other routes	128 (0.5%)	8 (0.1%)	1 (0.1%)	119 (0.7%)	0 (0.0%)	1 (0.1%)	15 (0.7%)	83 (0.7%)	5 (0.5%)	10 (1.2%)	6 (1.3%)	2 (0.0%)
Unknown	2,894 (11.2%)	1,049 (13.5%)	189 (18.4%)	1,656 (9.8%)	68 (17.4%)	169 (20.8%)	300 (13.8%)	1,030 (8.4%)	93 (8.5%)	95 (11.4%)	90 (19.4%)	5,590 (81.7%)
**Median CD4+ count, cells/µl (IQR)**	322 (146, 524)	364 (157, 577)	315 (128, 545)	304 (143, 500)	319 (154, 552)	311 (97, 499)	340 (108, 598)	305 (157, 492)	322 (150, 502)	166 (40, 362)	338 (155, 521)	302 (100, 518)
**CD4+ category**												
<200 CD4 cell count/µl	5,669 (22.0%)	1,569 (20.2%)	208 (20.3%)	3,892 (22.9%)	86 (22.0%)	175 (21.6%)	421 (19.3%)	2,786 (22.8%)	269 (24.6%)	260 (31.2%)	103 (22.2%)	227 (3.3%)

Variable	Total	Non-migrants	Migrants born in the EU/EEA	Migrants born out the EU/EEA	Western Europe	Central Europe	Eastern Europe	Sub-Saharan Africa	Latin America and Caribbean	South and South-east Asia	Other	Unknown
200 to < 350 CD4 cell count/µl	3,717 (14.4%)	970 (12.5%)	122 (11.9%)	2,625 (15.5%)	58 (14.8%)	84 (10.3%)	199 (9.1%)	2,069 (16.9%)	181 (16.5%)	97 (11.6%)	59 (12.7%)	127 (1.9%)
350 to < 500 CD4 cell count/µl	3,286 (12.8%)	1,030 (13.3%)	105 (10.2%)	2,151 (12.7%)	44 (11.3%)	91 (11.2%)	178 (8.2%)	1,653 (13.5%)	166 (15.2%)	55 (6.6%)	69 (14.9%)	100 (1.5%)
>= 500 CD4 cell count/µl	4,830 (18.8%)	1,745 (22.5%)	181 (17.6%)	2,904 (17.1%)	82 (21.0%)	116 (14.3%)	418 (19.2%)	2,102 (17.2%)	210 (19.2%)	73 (8.8%)	84 (18.1%)	173 (2.5%)
Unknown	8,249 (32.0%)	2,437 (31.4%)	410 (40.0%)	5,402 (31.8%)	121 (30.9%)	346 (42.6%)	960 (44.1%)	3,620 (29.6%)	269 (24.6%)	348 (41.8%)	148 (32.0%)	6,211 (90.8%)
**AIDS**	3,395 (13.2%)	1,114 (14.4%)	162 (15.8%)	2,119 (12.5%)	48 (12.3%)	142 (17.5%)	329 (15.1%)	1,370 (11.2%)	159 (14.5%)	186 (22.3%)	47 (10.2%)	178 (2.6%)
**Reporting EU/EEA region**												
Eastern EU/EEA countries	1,508 (5.9%)	1,206 (15.6%)	19 (1.9%)	283 (1.7%)	3 (0.8%)	9 (1.1%)	261 (12.0%)	10 (0.1%)	4 (0.4%)	13 (1.6%)	2 (0.4%)	647 (9.5%)
Southern EU/EEA countries	4,237 (16.5%)	1,914 (24.7%)	68 (6.6%)	2,255 (13.3%)	43 (11.0%)	144 (17.7%)	192 (8.8%)	1,701 (13.9%)	197 (18.0%)	31 (3.7%)	15 (3.2%)	314 (4.6%)
Western EU/EEA countries	17,833 (69.3%)	4,257 (54.9%)	861 (83.9%)	12,715 (74.9%)	313 (80.1%)	622 (76.6%)	1,552 (71.3%)	9,356 (76.5%)	855 (78.1%)	471 (56.5%)	407 (87.9%)	5,599 (81.9%)
Northern EU/EEA countries	2,173 (8.4%)	374 (4.8%)	78 (7.6%)	1,721 (10.1%)	32 (8.2%)	37 (4.6%)	171 (7.9%)	1,163 (9.5%)	39 (3.6%)	318 (38.2%)	39 (8.4%)	278 (4.1%)
**Men**	76,921	48,023	5,280	23,618	3,058	3,863	3,140	8,940	5,117	2,250	2,530	13,012
**Median age, years (IQR)**	37 (29, 47)	39 (30, 50)	35 (29, 44)	35 (28, 44)	39 (30, 48)	34 (28, 42)	38 (31, 44)	37 (28, 46)	31 (26, 38)	32 (27, 40)	35 (28, 44)	39 (30, 50)
**Age category**												
≤18	1,038 (1.3%)	418 (0.9%)	39 (0.7%)	581 (2.5%)	17 (0.6%)	24 (0.6%)	76 (2.4%)	408 (4.6%)	25 (0.5%)	26 (1.2%)	44 (1.7%)	231 (1.8%)
19-29	19,672 (25.6%)	11,438 (23.8%)	1,449 (27.4%)	6,785 (28.7%)	722 (23.6%)	1,149 (29.7%)	517 (16.5%)	2,141 (23.9%)	2,170 (42.4%)	803 (35.7%)	732 (28.9%)	2,634 (20.2%)
30-50	41,459 (53.9%)	24,939 (51.9%)	3,149 (59.6%)	13,371 (56.6%)	1,728 (56.5%)	2,306 (59.7%)	2,252 (71.7%)	4,998 (55.9%)	2,590 (50.6%)	1,228 (54.6%)	1,418 (56.0%)	7,087 (54.5%)
>50	14,659 (19.1%)	11,173 (23.3%)	636 (12.0%)	2,850 (12.1%)	587 (19.2%)	376 (9.7%)	291 (9.3%)	1,384 (15.5%)	329 (6.4%)	186 (8.3%)	333 (13.2%)	3,006 (23.1%)

Variable	Total	Non-migrants	Migrants born in the EU/EEA	Migrants born out the EU/EEA	Western Europe	Central Europe	Eastern Europe	Sub-Saharan Africa	Latin America and Caribbean	South and South-east Asia	Other	Unknown
Unknown	93 (0.1%)	55 (0.1%)	7 (0.1%)	31 (0.1%)	4 (0.1%)	8 (0.2%)	4 (0.1%)	9 (0.1%)	3 (0.1%)	7 (0.3%)	3 (0.1%)	54 (0.4%)
**Mode of transmission**												
Sex between men	45,612 (59.3%)	31,832 (66.3%)	3,338 (63.2%)	10,442 (44.2%)	2,165 (70.8%)	2,226 (57.6%)	840 (26.8%)	1,726 (19.3%)	3,987 (77.9%)	1,442 (64.1%)	1,394 (55.1%)	1,766 (13.6%)
Heterosexual transmission (men)	17,606 (22.9%)	8,239 (17.2%)	736 (13.9%)	8,631 (36.5%)	444 (14.5%)	558 (14.4%)	804 (25.6%)	5,959 (66.7%)	759 (14.8%)	283 (12.6%)	560 (22.1%)	918 (7.1%)
Injecting drug use	3,644 (4.7%)	2,124 (4.4%)	430 (8.1%)	1,090 (4.6%)	106 (3.5%)	311 (8.1%)	803 (25.6%)	50 (0.6%)	31 (0.6%)	126 (5.6%)	93 (3.7%)	380 (2.9%)
Mother to child transmission	302 (0.4%)	71 (0.1%)	14 (0.3%)	217 (0.9%)	7 (0.2%)	4 (0.1%)	55 (1.8%)	145 (1.6%)	2 (0.0%)	7 (0.3%)	11 (0.4%)	19 (0.1%)
Other routes	89 (0.1%)	26 (0.1%)	14 (0.3%)	49 (0.2%)	6 (0.2%)	12 (0.3%)	14 (0.4%)	23 (0.3%)	1 (0.0%)	3 (0.1%)	4 (0.2%)	3 (0.0%)
Unknown	9,668 (12.6%)	5,731 (11.9%)	748 (14.2%)	3,189 (13.5%)	330 (10.8%)	752 (19.5%)	624 (19.9%)	1,037 (11.6%)	337 (6.6%)	389 (17.3%)	468 (18.5%)	9,926 (76.3%)
**Median CD4+ count, cells/µl (IQR)**	358 (170, 549)	373 (179, 562)	385 (192, 582)	320 (150, 510)	395 (215, 593)	369 (160, 570)	361 (149, 568)	280 (127, 450)	367 (203, 550)	295 (127, 472)	358 (181, 547)	314 (123, 534)
**CD4+ category**												
<200 CD4 cell count/µl	15,286 (19.9%)	9,164 (19.1%)	922 (17.5%)	5,200 (22.0%)	511 (16.7%)	736 (19.1%)	513 (16.3%)	2,410 (27.0%)	934 (18.3%)	513 (22.8%)	505 (20.0%)	603 (4.6%)
200 to < 350 CD4 cell count/µl	11,071 (14.4%)	6,567 (13.7%)	684 (13.0%)	3,820 (16.2%)	432 (14.1%)	459 (11.9%)	320 (10.2%)	1,659 (18.6%)	874 (17.1%)	362 (16.1%)	398 (15.7%)	343 (2.6%)
350 to < 500 CD4 cell count/µl	11,171 (14.5%)	7,196 (15.0%)	760 (14.4%)	3,215 (13.6%)	451 (14.7%)	504 (13.0%)	312 (9.9%)	1,220 (13.6%)	837 (16.4%)	285 (12.7%)	366 (14.5%)	310 (2.4%)
>= 500 CD4 cell count/µl	16,593 (21.6%)	11,000 (22.9%)	1,239 (23.5%)	4,354 (18.4%)	797 (26.1%)	834 (21.6%)	566 (18.0%)	1,324 (14.8%)	1,184 (23.1%)	318 (14.1%)	570 (22.5%)	495 (3.8%)
Unknown	22,800 (29.6%)	14,096 (29.4%)	1,675 (31.7%)	7,029 (29.8%)	867 (28.4%)	1,330 (34.4%)	1,429 (45.5%)	2,327 (26.0%)	1,288 (25.2%)	772 (34.3%)	691 (27.3%)	11,261 (86.5%)
**AIDS**	10,627 (13.8%)	6,754 (14.1%)	679 (12.9%)	3,194 (13.5%)	355 (11.6%)	560 (14.5%)	434 (13.8%)	1,386 (15.5%)	465 (9.1%)	345 (15.3%)	328 (13.0%)	500 (3.8%)

Variable	Total	Non-migrants	Migrants born in the EU/EEA	Migrants born out the EU/EEA	Western Europe	Central Europe	Eastern Europe	Sub-Saharan Africa	Latin America and Caribbean	South and South-east Asia	Other	Unknown
**Reporting EU/EEA region**												
Eastern EU/EEA countries	4,422 (5.7%)	3,745 (7.8%)	221 (4.2%)	456 (1.9%)	32 (1.0%)	189 (4.9%)	357 (11.4%)	10 (0.1%)	33 (0.6%)	37 (1.6%)	19 (0.8%)	1,277 (9.8%)
Southern EU/EEA countries	13,344 (17.3%)	9,078 (18.9%)	323 (6.1%)	3,943 (16.7%)	408 (13.3%)	436 (11.3%)	316 (10.1%)	1,327 (14.8%)	1,407 (27.5%)	234 (10.4%)	138 (5.5%)	1,001 (7.7%)
Western EU/EEA countries	53,689 (69.8%)	32,678 (68.0%)	4,242 (80.3%)	16,769 (71.0%)	2,298 (75.1%)	2,896 (75.0%)	2,177 (69.3%)	6,813 (76.2%)	3,092 (60.4%)	1,608 (71.5%)	2,127 (84.1%)	10,090 (77.5%)
Northern EU/EEA countries	5,466 (7.1%)	2,522 (5.3%)	494 (9.4%)	2,450 (10.4%)	320 (10.5%)	342 (8.9%)	290 (9.2%)	790 (8.8%)	585 (11.4%)	371 (16.5%)	246 (9.7%)	644 (4.9%)

Table S4. Modified Poisson model stratified by sex. Prevalence ratios and 95% confidence intervals for all reported new HIV diagnoses (2014–2023), with an explanatory variable for migrants vs. non-migrants.

	PR[1]	95% CI[1]	PR[1]	95% CI[1]
	Men		Women	
**Migration status**				
Non-migrants	Ref.	Ref.	Ref.	Ref.
Migrants born in the EU/EEA	1.06	1.02, 1.10	1.25	1.15, 1.36
Migrants born out the EU/EEA	1.19	1.17, 1.22	1.31	1.26, 1.36
**Time period of diagnosis**				
Pre-COVID	Ref.	Ref.	Ref.	Ref.
Post-COVID	1.01	0.99, 1.03	0.98	0.96, 1.01
**Age group**				
≤18	Ref.	Ref.	Ref.	Ref.
19-29	1.27	1.09, 1.47	1.14	0.98, 1.33
30-50	1.85	1.59, 2.16	1.50	1.30, 1.74
>50	2.34	2.01, 2.72	1.78	1.53, 2.06
**Reporting EU/EEA region**				
Northern EU/EEA countries	Ref.	Ref.	Ref.	Ref.
Western EU/EEA countries	0.87	0.84, 0.90	0.88	0.84, 0.93
Eastern EU/EEA countries	0.98	0.94, 1.04	1.11	1.03, 1.20
Southern EU/EEA countries	1.09	1.06, 1.13	1.00	0.95, 1.06
**Mode of transmission**				
Heterosexual transmission	Ref.	Ref.	Ref.	Ref.
Injecting drug use	0.92	0.89, 0.96	0.90	0.82, 0.99
Sex between men	0.70	0.69, 0.72		
Mother to child transmission	1.86	1.52, 2.29	1.09	0.80, 1.48
Other routes	1.20	1.04, 1.39	0.99	0.84, 1.17

[1]PR = Prevalence Ratio, CI = Confidence Interval, Ref= Reference category. Note: For each stratum, the model estimates report the Prevalence Ratio (PR), obtained by exponentiating the β coefficients from the modified Poisson model estimation, along with the corresponding 95% confidence intervals (CIs). Countries excluded from the analyses were: Bulgaria, Croatia, Finland, Hungary, Italy, Lithuania, Malta, Poland, Romania, Slovenia and Spain because were unable to classify diagnoses as either new or previously positive.
